# Quality of Life of Cypriot Patients Suffering with Huntington’s Disease

**DOI:** 10.1371/currents.hd.270776c4fdd7776499dd45bf47049a75

**Published:** 2016-10-25

**Authors:** Eleni Varda, Christiana A. Demetriou, Alexandros Heraclides, Yiolanda P. Christou, Eleni Zamba-Papanicolaou

**Affiliations:** The Cyprus School of Molecular Medicine, The Cyprus Institute of Neurology and Genetics, Nicosia, Cyprus; The Cyprus School of Molecular Medicine, The Cyprus Institute of Neurology and Genetics, Nicosia, Cyprus; Neurology Clinic D, The Cyprus Institute of Neurology and Genetics, Nicosia, Cyprus; Department of Primary Care and Population Health, University of Nicosia Medical School, Nicosia, Cyprus; Neurology Clinic D, The Cyprus Institute of Neurology and Genetics, Nicosia, Cyprus; The Cyprus School of Molecular Medicine, The Cyprus Institute of Neurology and Genetics, Nicosia, Cyprus; Neurology Clinic D, The Cyprus Institute of Neurology and Genetics, Nicosia, Cyprus

## Abstract

Introduction: Huntington’s disease (HD) has profound motor, behavioural and cognitive symptoms. Despite the enormous burden of this disease on the quality of life (QoL) of patients and their families, there is very limited evidence on this topic. Considering the severity of HD patients, and the high prevalence in Cyprus more studies are needed to assess QoL among Cypriot patients, in order to improve our knowledge about their living conditions and to assist the management of this condition.

Project Aim: The aim of this cross-sectional study is to assess QoL among Cypriot patients with HD, using a standardized health-related QoL questionnaire.

Materials and Methods: A generic QoL questionnaire was used, namely EQ-5D, which is a standardised instrument for use as a measure of health outcomes and is applicable to a wide range of health conditions. The study was conducted with 34 patients, which represented 46% of the Cypriot HD patient population.

Results: Ability of patients to care for themselves and to carry out usual activities were reported to be most severely affected (37.5% and 40.6% replying “Severe Problems” respectively). Mobility and psychosocial well-being were also affected to a lesser extent (25.0% and 15.6% replying “Severe Problems”). Interestingly, in the anxiety/depression scale, 77.8% of asymptomatic patients reported “Some Problems”. Half of the patients did not experience pain or discomfort but 40.6% reported “Some Problems” and 6.3% reported “Severe Problems”. The Health Status as perceived by the patients was found to be moderately to severely affected.  In multivariate ordinal regression analyses, age at onset and disease duration significantly impacted on self-care. In addition, disease duration was significantly associated with mobility, self-care and usual activities scales. No significant determinants were evidenced for Pain/Discomfort and Anxiety/Depression. Lastly, age of onset was found to be the only significant determinant of the cumulative QoL score (Range=5-15).

Conclusions: Age at onset and disease duration were found to severely affect the QoL of Cypriot HD patients, and more specifically their mobility, ability to self-care and perform usual activities. The percentage of patients reporting “Some Problems” in the Pain/Discomfort category can be explained by the direct translation of the word as presented in the questionnaire, indicating the need for language specific instruments. Perhaps more noteworthy is the phychosocial burden on even asymptomatic patients, which needs to be acknowledged and managed to improve their quality of life.

## Introduction

Huntington’s disease (HD) is a fatal progressive neurodegenerative disease of the central nervous system (CNS), in which patients experience profound motor, behavioural and cognitive symptoms^1^. Despite the enormous burden of this disease on the quality of life (QoL) of patients and their families, there is very limited evidence on this topic.

HD is a devastating disorder and besides progressive chorea is also characterized by rigidity and dementia^2^. A wide range of psychiatric disturbances and behavioral problems is also associated with the disease, which include depressed mood, anxiety irritability, apathy and psychosis^3^. It is important to catalogue the entire range of possible symptoms because they have a substantial impact on the ability of individuals to go about their daily activities, and the disease is known to cause severe disabling and distress^4^.

The inheritance type of disease is autosomal dominant, i.e. children of HD gene carriers have a 50% chance of inheriting the gene^5^. The mean age at onset of symptoms is 30-50 years, and leads to death within 17–20 years 6, where the juvenile HD appears in individuals under the age of 20, and is usually transmitted paternally^7^.

The genetic mutation is determined in chromosome 4p16.3, which encodes the huntingtin protein (348-kDa). A genetic alteration in the HTT gene causes HD due to the increased number of repetitions of “triplet” nucleotides ‘Cytosine Adenine Guanine’ (CAG)^8^, which is associated with accumulation of an abnormal misfolded protein. This can impair cell function and lead to neuronal loss and it affects large number of pathophysiological pathways, i.e. a proteinaceous aggregation, which interferes with cellular trafficking^9^.

Presenting the phenotype of HD patients, HD belongs to the family of movement disorders, which can be divided into two categories; hyperkinesia, which is defined as involuntary movements, classically chorea or not rhythmic movements, and hypokinesia of voluntary and automated movements^10^. Motor impairment is usually amongst the first symptoms of HD patients, and in progressive stages is characterized by dystonia, rigidity and bradykinesia^5^.

Behavioural alterations in HD often cause the most distress both patients and their families and are therefore often central in the practical clinical management of patients^11^. The most common psychiatric symptoms, which occur as part of the disease, include depression, anxiety, apathy, irritability, and obsessive-compulsive disorder (OCD) (3). Moreover, recent findings from Wetzel et al.^12^ show suicide rates were reported to be 9.5% in HD individuals, and suicidal ideation 26.5%. One of the leading causes of death of HD patients is suicide, another being pneumonia^13^.

The final important aspect of HD in patients is cognitive impairment. HD is associated with significant memory decline in early stages, and even poorer presentation in later stages, compared to healthy individuals 14 ^,^ 15.

HD occurs in all racial groups but a higher prevalence was observed in Europe, North America, and Australia with 5.70 cases per 100.000, with a much lower in prevalence in Asia of 0.40 per 100.000 16. Prevalence of HD in Cyprus in 2012 was 7.22 per 100,000, while the incidence was 0.62 per year. Prevalence in Cyprus is therefore higher than the respective worldwide figure, although the sample size is too small to make definitive statements.

Quality of Life (QoL) measures provide an effort to improve care. Clinical research sometimes considers these as outcome measures, but clinical practice has not, thus far, made significant use of them (Higginson and Carr 2001). QoL has potential uses in aiding routine clinical practice. They can be used to obtain a ranking of problems according to severity, maintain focus on patients’ main complaints, and identify less obvious issues, particularly psychological ones, improving clinical cooperation and monitoring treatment performance. They can also be used in clinical audit and in clinical governance^17^. Overall, QoL measures can be a predictor of treatment success, and several studies have shown that factors such as QoL, physical well-being, mood and pain are of prognostic importance^18^.

There is an increasing body of research associated with the negative impact of HD in the QoL of patients. The majority of studies conclude that HD has an adverse effect on patients' physical and psychosocial well-being, where the effect on the latter is greater^19^^,^ 20 ^,^ 21. A recent study has demonstrated that people with progressive neurological disorders, such as Alzheimer Disease, Parkinson Disease or HD, suffer from negative mood swings and have lower QoL scores. The findings show that HD patients exhibited the most severe illness-related symptoms, and the greatest effects on mood and their QoL. In particular, HD patients had the least control over their bodily functions, found the greatest difficulty in tasks requiring cognitive functions, and experienced the greatest number of psychological symptoms, as well as high levels of confusion^21^.

Even compared to other neurodegenerative disorders, neuropsychiatric symptoms in HD have been found to have a much greater effect on QoL. In particular, behavioural alteration is prevalent and psychopathology is affected more severely^21^^,^ 22. Considering the severity of HD, the fact that currently there is no cure for the disease, and the high prevalence of HD in Cyprus, more studies are needed to assess QoL among Cypriot patients, in order to improve our knowledge about their living conditions and to assist the management of this condition.

The aim of this cross-sectional study was to assess QoL among Cypriot patients with Huntington disease, using a standardized health-related quality of life questionnaire. The specific objectives of this study were to assess the quality of life of Huntington disease patients visiting the Cyprus Institute of Neurology and Genetics for follow-up and treatment and to investigate socio-demographic and clinical determinants of quality of life among these patients (i.e. gender, current age, age at onset of disease, age tested, disease status, parent-of-origin and number of repeats in HD allele).

## Materials and Methods

**Study Design and recruitment of participants**

The present study took place at Neurology Clinic D, at the Cyprus Institute of Neurology and Genetics (CING). Ethical approval was granted by the Cyprus National Bioethics Committee (ΕΕΒΚ ΕΠ 2013.01.06).

The target sample for the project comprised of all alive HD patients registered at CING (n=62). From those, it was possible to contact 37 participants. The remaining 25 were either too severely disabled to come to CING for completion of the questionnaire or their treating physicians suggested that these particular patients had refused any contact from CING previously. Out of those 37, 3 refused to participate, thus the total number of participants for the current study was 34. Participants were recruited at CING during routine visits, and after having signed a Consent Form .

The only inclusion criteria for patients were to have a confirmed diagnosis of HD and to have given consent for participation, via the aforementioned Consent Form. Participants were already aware of their condition during data collection.

Once participants had read the information in the Consent Form and signed the forms, they were asked to answer the EQ-5D Questionnaire, in order to assess their QoL. All questionnaires were anonymized using the patient’s unique CING medical record number, which was written on the questionnaire.

For patients at advanced stages of HD who may not have been in a position to read and comprehend the Consent Form and the Questionnaire due to cognitive impairment, a proxy version of the questionnaire was given to a proxy for completion.

HD patients who were unable to visit the CING either due to the severity of their illness or due to personal reasons were asked to participate in a telephone interview by their doctors. Consent Forms were given to them upon visiting the CING for their routine appointment.

**Assessment of Quality of Life**

A generic QoL questionnaire, the EQ-5D, was used, which is a standardised instrument for use as a measure of health outcome and is applicable to a wide range of health conditions. The type of EQ-5D questionnaire that was used was the EQ-5D-3L, as this was the only version translated and validated in the Greek language. EQ-5D-3L consists of two sections; the EQ-5D descriptive system and the EQ VAS.

**Scoring the EQ-5D Descriptive System**

The first section in the questionnaire, the EQ-5D descriptive system, comprises 5 dimensions: mobility, self-care (intended to capture the ability of patients to, e.g. wash or dress their selves), usual activities (intended to capture the ability of patients to work, study, perform housework and engage in family or leisure activities), pain/discomfort and anxiety/depression (hereafter ‘’5D’’). Each dimension has three levels (hereafter ‘’3L’’), which are defined as: no problems (level 1), some problems (level 2), and severe problems (level 3). The respondent was asked to indicate his/her health state by ticking in the box against the most appropriate statement in each of the 5 dimensions. This decision resulted in a 1-digit number expressing the level selected for that dimension. It should be noted that only one response was accepted for each dimension.

A cumulative QoL score was constructed by adding up all the values of the EQ-5D Descriptive System state (mobility, self-care, usual activities, pain/ discomfort and anxiety/depression). The sumulative QoL score could range from 5 (no problems in any scale and thus a good QoL) to 15 (severe problems in all scales and thus a bad QoL).

**Scoring the EQ VAS**

The second section, the EQ VAS, uses a visual analogue scale to capture the respondent’s self-assessment of their health on a continuous scale, where the higher endpoint is labelled ‘best imaginable health state’ and the lower endpoint reflects the ‘worst imaginable health state’. This information was used as a self-rated quantitative measure of health.

**Statistical Analysis**

The statistical software programme STATA version 12 SE used to perform the required descriptive and inferential analysis.

The main characteristics of participants including demographic (gender, disease status and parent-of-origin) and clinical characteristics (current age, age at onset, age tested, years since onset and number of repeats of mutant HD allele) were first examined. In addition, the variables for each of the five dimensions of the EQ-5D Descriptive System were analysed, along with the cumulative QoL score and the EQ VAS score.

Univariate associations between the different demographic and clinical characteristics and the QoL measures were assessed using one-way ANOVA, Kruskal-Wallis, and Fisher’s exact tests depending on the nature of the variables examined.

Lastly, multivariate models were used to assess the combined effect of demographic and clinical characteristics on QoL outcomes. Given the ordered nature of the responses in each outcome of the EQ-5D Descriptive System, ordinal logistic regression was used. The cumulative QoL score and the EQ-VAS scores were categorized into 2 groups (above/below the median) and were analysed using logistic regression, because of their non-linear nature and the failure of several common transformations to normalize them. Because of collinearity between current age and years since onset (r=0.4305, p=0.005), only years since onset was included in the models to reflect disease duration. Keeping current age instead of years since onset, did not alter the results indicating that both variables equally well reflect disease duration. In addition, parental mode of inheritance and gender were not significantly associated with any outcome and were thus removed from the models to achieve a better fit. Lastly, because the majority of asymptomatic patients reported No Problems in all scales, the multivariate analyses were only performed on symptomatic patients (n=23).

## Results

**Demographic and Clinical Characteristics of Cypriot HD patients**

All patients recruited met the inclusion criteria. However, two patients were unable to complete the questionnaire and a proxy version of the questionnaire was filled in by their spouses. Due to the likely heterogeneity between patient and carer perspectives, especially when patients are severely functionally impaired, these two patients were excluded from further analyses. Table 1 summarizes the demographic and clinical characteristics of the remaining 32 patients who took part in the study. The majority of the patients were women 62.5% (n=20), while men were 37.5% (n=12). The median age of the patients at the time of the study was 52.5 years.

Among these 32 patients, 72% were symptomatic HD patients, while the other 28% were asymptomatic HD patients. For the 23 symptomatic patients, the median age at onset was found to be 43 years, which was close to the median age of testing of 41.5 years. The majority of the patients, 64.5%, had inherited the HD gene maternally. Finally, of the total 32 patients that were genetically tested, the median number of the normal allele was 17 CAG repeats, whereas the median number of the mutant allele was 43 repeats.

**Figure:**
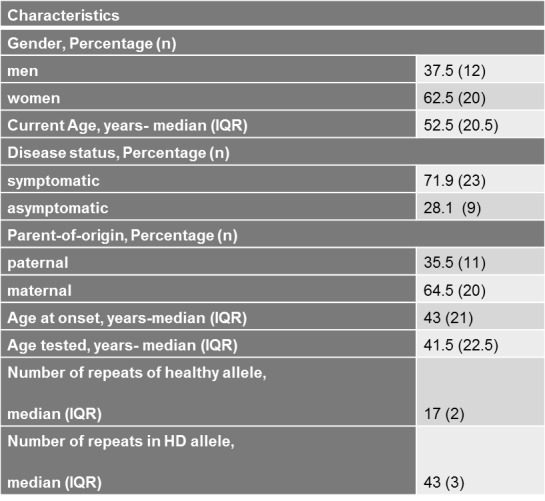
**Table 1. ** Baseline Patients’ Demographic and Clinical Characteristics of Cypriot HD patients

**Quality of Life among Cypriot HD patients:** **The EQ-5D Descriptive System among Cypriot HD patients**

The results of the EQ-5D Descriptive System, which was comprised of five dimensions each of which has three levels, are presented in Table 2.

Over 40% of the patients had mild problems with their motor function, while a lower percentage (25.00%) had experienced motor impairment (i.e. severe problems). Surprisingly, the vast majority patients (81.25%) were split equally between reporting no problems and severe problems with their self-care. However, the majority of patients (40.63%) had severe problems in performing their usual activities, which included work, study, housework, family or leisure-related activities. On the other hand, 93.75% had either reported some or no pain. Lastly, the majority of the HD patients (>70%), had experienced some anxiety or depression, with slightly more than 15% experiencing severe problems.

**Figure:**
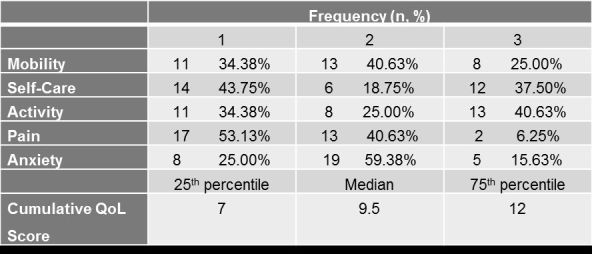
**Table 2.** Frequency Proportions for each score of the EQ-5D Descriptive System among Cypriot HD patients

Looking at the cumulative EQ-5D Quality of Life score, 50% of HD patients had a score below 9.5, and 25% of HD patients had a score above 12, which indicates a poor QoL.

**The EQ-VAS scores of Cypriot HD patients, a self-assessed health measure:**
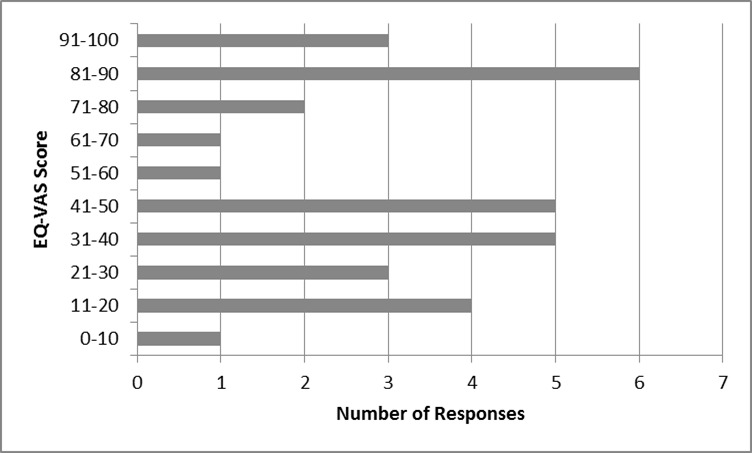

**Quality of Life among Cypriot HD patients: The EQ- VAS score among Cypriot HD patients**

The EQ-VAS score indicated that contrary to what was expected, a large number of patients reported a perfect health status. On the contrary, one patient reported an EQ-VAS of lower than 10%. The median (IQR) was found to be 50 (60).

**Determinants of Quality of Life among HD patients :** **Univariate analyses per scale outcome of the EQ-5D Descriptive System**

Univariate associations between the different scales of the EQ-5D Descriptive System and different demographic (gender, current age, disease status and parent-of-origin) and clinical characteristics (age at onset, age tested, and number of repeats of mutant HD allele) are shown in Tables 3-7.

Only current age, age tested, and disease status were significantly associated with the mobility score (Table 3). The severity of problems was positively associated with age (p=0.0007) and age tested (0.0015). Also, as expected, asymptomatic patients all reported no problems with their mobility in contrast to symptomatic patients (p<0.0001).

**Figure:**
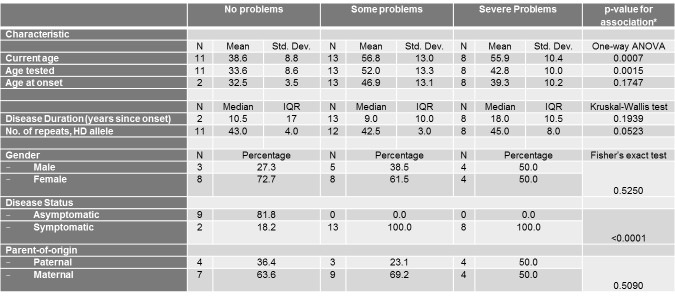
**Table 3. ** The association between the mobility Scale of the EQ-5D Descriptive System and different socio-demographic and clinical characteristics

**Figure:**
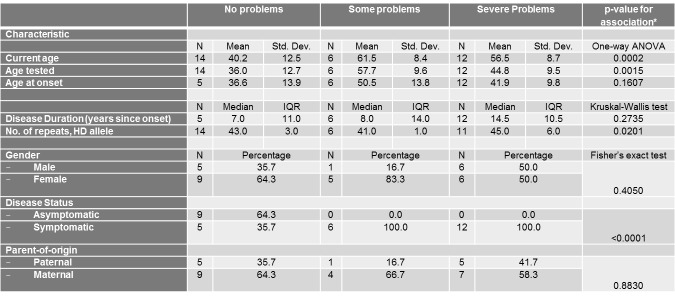
**Table 4. **The association between the self-care Scale of the EQ-5D Descriptive System and different socio-demographic and clinical characteristics

**Figure:**
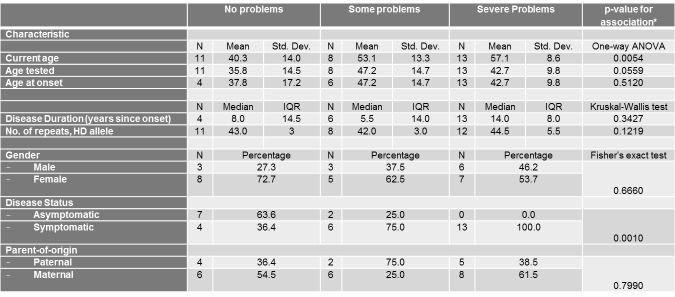
**Table 5. **The association between the usual activities Scale of the EQ-5D Descriptive System and different socio-demographic and clinical characteristics

Similarly, current age and age tested were significantly associated with the self-care score (Table 4) and current age was also associated with the usual activities score (Table 5). The severity of problems was positively associated with current age (p for self-care=0.0002, p for usual activities=0.0054) and age tested (p for self-care=0.0015). Also, as expected, disease status was associated with self-care and usual activities scores (p for self-care<0.0001, p for usual activities<0.0001). Asymptomatic patients all reported no problems with self-care and all but 2 asymptomatic patients reported no problems with usual activities. Self-care score was also positively and significantly associated with number of repeats in HD allele (p=0.0201).

With respect to Pain/Discomfort and Anxiety/Depression, no demographic or clinical characteristic demonstrated significant associations (Tables 6-7).

**Figure:**
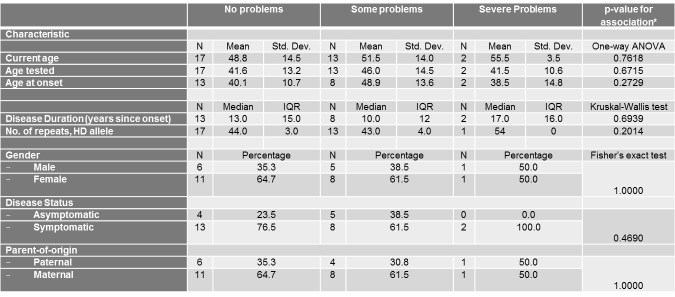
**Table 6. **The association between the pain/discomfort Scale of the EQ-5D Descriptive System and different socio-demographic and clinical characteristics

**Figure:**
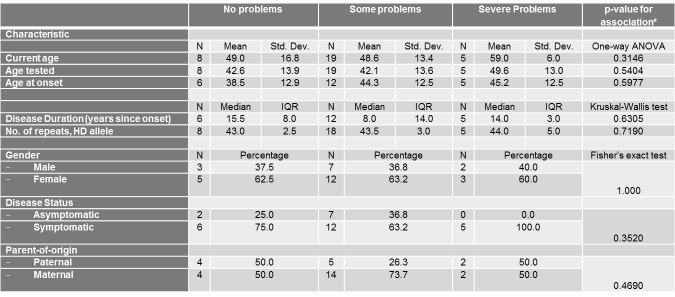
**Table 7. **The association between the anxiety/depression Scale of the EQ-5D Descriptive System and different socio-demographic and clinical characteristics

**Determinants of Quality of Life among HD patients : Multivariate analyses per scale outcome of the EQ-5D Descriptive System**

From multivariate analyses, it became evident that age of onset was independently and significantly associated with the self-care scale. For each year older a subject was at disease onset, they had 46% increased odds of reporting some or severe self-care problems compared to no problems. The significance of age at onset on the self-care scale may reflect the influence of current age since these two variables were highly collinear.

Disease duration was perhaps the most important determinant, since it was significantly associated with the mobility, self-care and usual activities scales. For each additional year of disease duration, patients were 19%, 33% and 22% more likely to report some or severe problems for the mobility, self-care and usual activities scales respectively. For these three scales, age tested did not retain the significance demonstrated in the univariate analyses probably due to the stronger effect of disease duration on the scales.

Similar to the univariate analyses, for Pain/Discomfort and Anxiety/Depression scales, no demographic or clinical characteristic demonstrated significant associations (Table 8).

**Figure:**
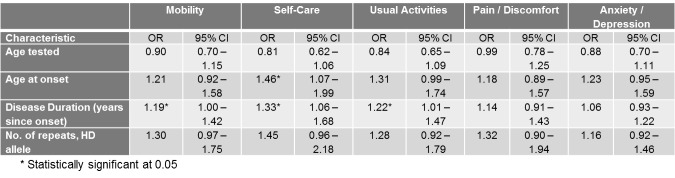
**Table 8. **Multivariate analyses using ordinal logistic regression between demographic and clinical characteristics and each EQ-5D Descriptive System outcome

**Determinants of Quality of Life among HD patients :** **Cumulative Quality of Life Score**

Of the independent demographic and clinical characteristics examined, only current age and age tested were significantly associated with having a cumulative QoL index score above median. However, in the multivariate model, where current age was not retained, and contrary to what was expected from the multivariate analyses of the individual EQ-5D Descriptive System scales, only age at onset was statistically significantly associated with the cumulative QoL. More specifically, for each one-year increase in age at onset, a subject had 85% increased odds of reporting a QoL score above 9.5, which translates into worse quality of life.

**Figure:**
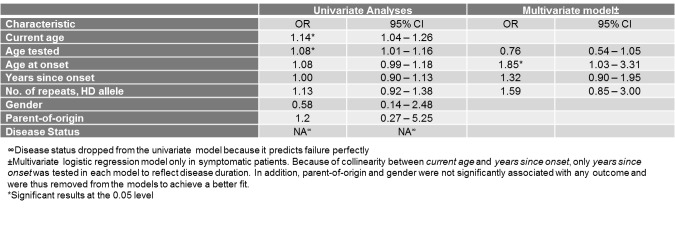
**Table 9. **Univariate and multivariate analyses between demographic and clinical characteristics and the cumulative Quality of Life score

**Determinants of Quality of Life among HD patients : The EQ-vas scale**

In univariate analyses, current age and age tested were significantly and negatively associated with having an EQ-vas score above median. For each one-year increase in current age, each individual had 13% decreased odds of reporting an EQ-vas score above 50, demonstrating decreased self-assessment of their health. The respective percentage for each one-year increase in age tested was 9%. Most importantly, symptomatic disease status also decreased by 95% the odds of reporting an EQ-vas score above median. However, in the multivariate model in symptomatic patients, none of these characteristics retained their statistical significance. This might reflect that onset of disease might in fact have a greater impact on health status self-assessment than do mobility, self-care, and usual activity problems which are associated with age at onset and disease duration.

**Figure:**
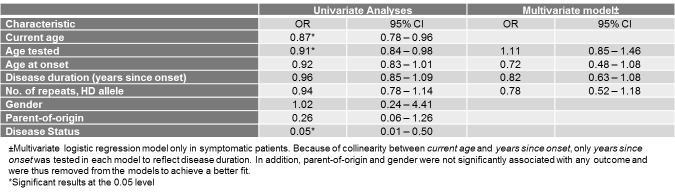
**Table 10. **Univariate and multivariate analyses between demographic and clinical characteristics and the EQ-vas score

## Discussion

**Overall Quality of Life**

This analysis determined that over 40% of the Cypriot HD patients showed particularly severe impairment in their ‘self-care’ competence and their ability perform their ‘usual activities’ . These results are in agreement with the study of Helder et al. 2001^19^, which using a different questionnaire on 77 Dutch patients, concluded that there was severe impact of HD on the ability of patients to carry out their usual activities.

Helder’s results also showed that a large percentage of the patients reported difficulty in maintaining productive employment due to deterioration in alertness faculties (e.g., forgetfulness, attentional and problem-solving deficits) and problems in the physical domain, which contributed to their inability to continue working. A significant percentage of patients also reported severe impairment in the categories of “home management” and “recreation and pastimes”. However in the study of Helder et al., there was a small percentage of patients reporting problems with their ability to eat (which can be considered as a proxy for self-care in the EQ-5D system), which is in contrast to the study of the Cypriot patients’ QoL. As in this study, other studies have shown that the impact of HD on the usual activities of patients and on their self-care abilities becomes more severe with progressive stages of the disease^23^.

In this study, the majority of the patients also reported moderate problems in their mobility status, as expected. HD belongs to the family of movement disorders 10 and in progressive stages is characterized by dystonia, rigidity and bradykinesia^5^. In fact, motor impairment is a profound symptom of HD patients. More specifically, it includes falls, gait and sleep disturbances^24^^,^ 25. These results are in agreement with several other studies assessing the motor impairment by questionnaire in HD patients, which concluded that motor symptoms are negatively associated with the QoL of HD patients 19 ^,^ 20 ^,^ 24.

However, in the above mentioned studies 19 20 24 , the major factor implicated in the poor QoL of patients is the psychological factor. More specifically, these studies concluded that HD patients showed more severe impairment on the psychosocial domains than in the physical domains. In particularly, they assessed determinants such as ‘depressive mood’, ‘emotional behaviour’, ‘alertness behaviour’ and ‘psychosocial dimension’. This is in agreement with our study since a high percentage of the Cypriot patients (over 60%) reported moderate anxiety/depression status. This is an expected finding in the context of HD studies, since the behavioural alteration in HD patients is a common symptom^11^. Moreover, a study from McCabe et al. (2009)^21^, assessing the QoL of among three motor diseases (in particularly, AD, PD and HD), showed that HD patients experienced the greatest effects in their mood and QoL. Interestingly, even 77.8% of asymptomatic patients reported a moderate anxiety/depression status indicating the psychosocial burden of the disease even before its onset.

Furthermore, less than 6% of Cypriot patients reported severe ‘pain or discomfort’ problems, while approximately 45% patients reported moderate problems (Figure 4). Regarding pain effects, a study from Tomaaso et al. (2011)^26^, which evaluated pain perception in HD patients, concluded that pain is not a common symptom in HD. This is confirmed with other studies, were bodily pain was negatively related to the illness perceptions of HD patients^27^^,^ 28. This symptom is in contrast to other neurodegenerative diseases, such as PD^29^.

Regarding the extent of ‘discomfort’, it is important to mention that the word ‘discomfort’ has an ambiguous meaning in Greek. The direct translation of the word as presented in the questionnaire may have influenced the response of the participants because in the Greek language, the word points towards both physical and mental discomfort. This is in contrast to the English word, which reflects physical uneasiness. This can perhaps explain some of the variation in the percentages of patients selecting moderate and severe problems in this category. Since pain is not common symptom of the disease, and as per the Cypriot patients’ responses, the state ‘discomfort’ should be consider as both mental and physical^20^.

Assuming that, to the patients’ understanding, the word ‘discomfort’ indicates both mental and physical discomfort in Greek, this subsection further confirms that HD has an adverse effect on Cypriot patients' psychosocial well-being. This result could be due to the fact that psychosocial well-being is not only determined by illness-related factors, such as motor or cognitive disabilities, but primarily by psychological concomitants of that illness, including the way in which patients cope with their disease.

The median Cumulative QoL was found to be 10 indicating moderate problems in all scales and thus a moderate QoL. This is in agreement with similar studies, conducted, however, using different questionnaires, which concluded that HD has a severe impact on patients' physical and psychosocial well-being (i.e. depressive mood, anxiety), with the latter being more prominent^19^^,^ 20 ^,^ 24.

On the other hand, the EQ-vas score, which can be considered as an aggregate self-reported health status of the patients, ranged from average to poor (Figure 8). The EQ-vas score amongst Cypriot patients does not seem to follow a normal distribution, since a large number of patients reported a perfect health status. It is important to note that in this study, although there was proxy version of the questionnaire, which was given to patients’ relatives for completion in the case where patients had some cognitive impairment, there were also participants who answered the questionnaire and had some form of cognitive disability. This was confirmed by the patients’ doctors. It resulted in some patients, who were already in later stages of the disease, bound to a wheelchair, reporting a Health Status of 100%. This is possible because HD patients in progressive stages, who had experienced severe mobility impairment, are likely to experience cognitive impairment as well^23^. This has occurred because in our study we did not use a ‘mini mental stage examination’, which is commonly used in neurodegenerative studies, including HD^30^^,^ 31.

**Determinants of Quality of Life - Demographic Determinants**

**Gender**

Our sample was comprised of more women than men (Table 1), but this is unlikely to have significantly influenced the results, given what is known about HD and, in particular, also QoL gender comparison. Starting with the effect of gender examined in the study, we related it to the categories of the EQ-5D Descriptive System, Cumulative QoL and the EQ-vas scale, however, no association was found to be significant, as was expected from a study from Mahant et al. (2003)^32^, which evaluated the clinical correlation and the progression of the HD, and did not find any association between the rate of progression of the disease (thus, the poorest QoL) and the sex of the affected individual.

**Current Age**

Regarding the age of the patients, the association of age with mobility, self-care and usual activities status was positive and significant in univariate tests.

This is in agreement with the Ho and Hocaoglu (2011)^23^ study, which assessed the phases and the stages of HD from patients (ages between 30-89 years old), and found that as the stages of the disease progressed, the symptoms of the disease became more noticeable. In particular, in the last stage of the disease (older patients), the issues raised were physical or functional (i.e. difficulties with ambulation, swallowing, sleeping, speaking, writing and dressing). Generally, as the individual aged and the disease progressed, the symptoms of the disease became more evident and this has as an impact on Health Status and the QoL of the patient. This can be seen in our findings also, as the EQ-vas scale analysis and the Cumulative QoL score analysis showed significant associations between the current age of the patients, indicating that both QoL and overall health status deteriorates with age.

There was no association between the current age of the patients and the presence of psychological problems. This indicates that psychological issues in HD are independent from the age of the patient. This also has an intuitive explanation, as even in pre-symptomatic stages (i.e. patients who are younger than 40 years old, excluding juvenile cases) patients can feel stress for the extent of the detrimental effects of the disease^33^.

However, multivariate models suggested that current age might not be an independent predictor of QoL but its effect on QoL might instead be a reflection of disease duration, as discussed further down.

****Determinants of Quality of Life**** **** **-** **Clinical** **Determinants**

**Disease status (symptomatic and asymptomatic)**

The study determined that there was a significant statistical association between most categories of the EQ-5D Descriptive System and disease status. However, for ‘pain/discomfort’ and ‘anxiety/depression’, the difference in status between symptomatic and asymptomatic patients was not significant.

As discussed above, it is possible that Cypriot patients perceived the word ‘discomfort’ to point to both mental and physical discomfort, therefore the result shows that even asymptomatic gene carriers had a psychological alteration, although they had not yet experienced any symptoms of the disease. Generally, pre-symptomatic HD patients participating in other HD studies had psychosocial QoL issues relevant to this subgroup as well, whereas physical, functional and cognitive issues hardly featured in HD gene carriers^33^^,^ 34.

Overall, in the EQ-vas scale and the Cumulative QoL score, asymptomatic patients reported better QoL and better Health Status, respectively, in contrast to symptomatic patients, and the differences in these scores was statistically significant.

**Parent-of-origin (paternal and maternal)**

The sample of the study comprised of more patients who had inherited HD maternally, rather than paternally. However, the parent-of-origin was not found to be a statistically significant factor in determining the extent of the problems considered in the EQ-5D descriptive system. We are not aware of any studies considering the association of the parent-of-origin transmission and the QoL of HD patients. From our results, one can postulate that the parent-of-origin is not associated with the QoL of the patients, but further research on a larger sample size of HD patients is required, in order to confirm this finding.

**Age at onset and Age tested**

Age at onset was another determinant considered, and the results show that of the patients reporting severe problems in the 5 EQ-5D Descriptive System categories, the majority had an age at onset above 40 years old. The same trend was evident for patients reporting a cumulative QoL above median. However, statistical associations between age at onset and the determinants of the EQ-5D Descriptive System (with the exception of self-care) and the EQ-vas scale were not found to be significant in multivariate analyses.

Interestingly, patients who had reported severe problems in the majority of the EQ-5D Descriptive, were younger at onset than patients who had reported moderate problems, reflecting the association between number of repeats of the mutant allele, earlier age at onset and worse disease progression. Therefore, the unexpected positive association between age of onset and reporting moderate or severe problems in the 5 EQ-5D Descriptive System categories or reporting a higher cumulative QoL is most likely driven by the larger number of patients with a higher age-of-onset that reported “Some Problems”.

In contrast to age at onset, age tested was a significant determinant of mobility and self-care scale scores. However, similar to the results considering the age at onset of the disease, patients reporting severe problems in the mobility, self- care and usual activities status of the EQ-5D Descriptive System were generally over 40 years old and patients tested between the ages of 40-48 years old, reported severe problems, whereas patients tested when they were over 49 old years reported moderate problems, a difference which was statistically significant. These results are in agreement with another large study, which found that the rate of HD progression was more rapid with younger age at onset, in particular with issues such as motor impairment (dystonia) and the rate of cognitive and functional progression^32^.

When both variables were entered in multivariate regression models, age tested did not retain its significance, and instead, age at onset was positively and significantly associated with self-care problems compared to no problems. This indicates that of the two ages, age at onset is most influential in determining quality of life.

**Disease Duration (years since onset)**

Years since onset was used in univariate and multivariate analyses as an indication of disease duration. Even though disease duration was not significantly independently associated with the five EQ-5D scales, in multivariate analyses in symptomatic patients, correcting for age at onset and age tested, disease duration was significantly and positively associated with worsening problems in mobility, self-care and usual activities. This makes disease duration perhaps the most influential predictor of QoL, after accounting for all other demographic and clinical characteristics. This was also evident in multivariate analyses on the cumulative QoL where years since onset was the only significant predictor.

In related research, patients with either juvenile onset of disease or late onset of symptoms had significantly shorter disease duration than those who had onset in mid-life (onset 20-49). The course of HD is probably shorter in the older onset of HD, due to other unrelated conditions which can shorten life expectancy^35^. These findings might explain why disease duration was significantly associated with QoL only after adjusting for age at onset.

Surprisingly, the impact of disease-duration was not evident in multivariate analyses on the cumulative QoL where, instead, age at onset was the only significant predictor. Larger subject numbers are needed to delineate the interaction between age of onset and disease duration on the QoL and fully explain these findings.

**Number of repeats in HD allele**

For patients who reported severe problems, the number of repeats in their mutant HD allele usually exceeded 45 repetitions. More specifically, the correlation between the number of repeats and the severity of issues in mobility, the self-care and pain or discomfort status was positive, despite being non-significant with the exception of the self-care scale.

These results are in agreement with a larger related study considering the association of the number of CAG repeats and the clinical progression of more than 500 HD patients^31^. The result in that study was that the number of repeats was a small but significant predictor of progression rates of HD (measured using neurological signs, motor impairment, cognition and daily function).

**Strengths and Limitations**

This study assessed, for the first time, the QoL of HD patients in Cyprus and aimed to identify some of the major factors affecting QoL. This study can be regarded as the first step in an approach to improving living conditions among these patients, with informed and targeted health promotion programmes. The study invited all alive patients with Huntington’s disease who were physically and cognitively able to consent to and participate in the study. In addition, all demographic and clinical characteristics were extracted from patient files, instead of obtained through self-report, minimizing information bias.

Despite the strengths of the study described above, it also had some limitations. First, the sample size of the study was too small. Of the Cypriot registered HD patients, only the 42.6 % took part, while the remaining 57.4% did not participate for several reasons. Some were either too severely disabled to come to CING or be contacted for completion of the questionnaire, or because their treating physicians suggested that particular patients had refused contact from CING previously. Not considering these patients is a limitation, because the study may not be capturing the true extent of the issues considered for HD patients, since the most severely affected patients were not part of the study. This is particularly true following the exclusion of the small number of carer/proxy reports for the cognitively impaired, advanced-stage HD patients. Of the other patients, some were unable to attend, due to personal reasons, such as professional obligations or because they were living in a ward.

Secondly, some patients had reported overly positive health states, which may have been related to some mental impairment. A mini mental state of examination was required since it is a reliable method to assess the cognitive disability of the patients and is commonly uses in neurological studies, including HD^30^^,^ 31.

Finally, the Greek translation of the questionnaire may not have been the most accurate in carrying across the meaning of some words. For example, the word ‘discomfort’ in Greek points not only to the physical domain (such pain), but also to the mental domain. This was confusing for the Cypriot patients.

**Future work**

A first extension of this work would be to further the investigation in order to assess the QoL of the remaining Cypriot patients, who did not have the opportunity or the capacity to take part. Obtaining evidence from the entire Cypriot patient population is important so that we can characterise definitively the aspects affecting QoL. Further work in HD would necessarily include a short mental test prior to the completion of questionnaires, to determine whether the patient can accurately self-assess their physical mental state.

In addition, the study used a generic questionnaire assessing the QoL of HD patients. Once the HD specific questionnaire is approved and a Greek translation is available, it would be useful to see how the new questionnaire could add to the pool of evidence regarding HD. HD is a multi-faceted disease, and the additional considerations of an HD-specific questionnaire will be useful in this aspect.

## Conclusions

The study assessed, for the first time, the QoL of HD patients in Cyprus and demonstrated that QoL in the Cypriot HD patient population was moderately to severely affected by the disease. The disease was most frequently found to affect the ability of Cypriot patients to carry out their usual, day-to-day activities and to care for themselves. The psychological state was found to play a crucial role in the QoL of HD patients, since the majority of the patients, including pre-symptomatic ones, reported moderate anxiety and depression. In terms of the physical domain, a large number of HD patients reported moderate problems in their mobility status. The study did not identify pain as being a symptom in the tested population, although discomfort was prevalent. The overall Health Status of the patients was defined as average to poor, whereas the Cumulative QoL score indicated moderate issues with QoL. Disease duration was evidenced as perhaps the most important determinant of QoL, after accounting for all relevant demographic and clinical characteristics, including disease status.

The results of this study could potentially be utilized for improving the quality of management of HD in Cyprus, in both symptomatic and asymptomatic patients. They can be utilized by clinics and patient support groups in order to better support, empower and care for HD patients and their families.

## Data Availability Statement

The authors provide detailed data regarding quality of life responses in Tables 3-7. All statistics were derived from information in these tables. Unfortunately, more detailed information and specific subject characteristics cannot be made publicly available due to ethical restrictions from the Cyprus National Bioethics Committee. For further information regarding data availability please contact christianad@cing.ac.cy.

## Competing Interests

The authors have no financial or non-financial competing interests to declare.

## Corresponding Authors

Eleni Zamba-Papanicolaou (ezamba@cing.ac.cy) and Christiana A. Demetriou (christianad@cing.ac.cy)
